# From collection to correction: Can serial 24‐hour urine collections demonstrate improved urinary stone parameters?

**DOI:** 10.1002/bco2.70070

**Published:** 2025-08-26

**Authors:** Daniel Jhang, Jason Groegler, Akin S. Amasyali, Hyukje Sung, Matthew Buell, Jersey Castillo, Elizabeth A. Baldwin, Mohamed Keheila, Zhamshid Okhunov, D. Duane Baldwin

**Affiliations:** ^1^ Department of Urology Loma Linda University Health Loma Linda CA USA

**Keywords:** nephrolithiasis, urinalysis, urinary calculi, urine, urine specimen collection, urolithiasis

## Abstract

**Objectives:**

To determine whether serial 24‐hour urine collections from the same patient over time result in improved stone risk parameters.

**Patients and Methods:**

Using a 24‐hour urinalysis database, 1832 tests from 688 patients collected over a 10‐year period were retrospectively reviewed. Patients included in the analysis had a minimum follow‐up of 2 years and at least three 24‐hour urine collections. Changes in risk parameters were evaluated over time with each patient acting as their own control. Statistical analysis was performed using repeated measures ANOVA with a Greenhouse–Geisser correction and post‐hoc analysis with Bonferroni correction. Significance level was set as p < 0.05.

**Results:**

A total of 225 patients had at least three 24‐hour urine collections, of which 48% were female. From the first to the second 24‐hour urine collections, volume and supersaturation of CaOx, CaP and UA all significantly improved (p < 0.05). From the second to the third collection, only the supersaturation of CaP significantly improved (p < 0.05). Approximately half of the patients continued to see improvement in stone risk parameters between the second and third urine collection.

**Conclusion:**

Serial 24‐hour urine collections performed at 6‐month or greater intervals were significantly associated with improvements in stone risk parameters. This study suggests that serial collections can aid in the correction of urinary stone parameters and should be considered in active stone formers.

## 1 INTRODUCTION

Urolithiasis is a significant source of patient morbidity and economic burden, given its increasing prevalence, cost and high recurrence rate. The data from the 2015–2018 National Health and Nutrition Examination Surveys showed an 11% stone prevalence, which was predicted to increase.^1^ Additionally, the recurrence rate approaches 50% at 10 years after the first stone episode.^2^ In 2012, the yearly cost of treating kidney stones was estimated at over $10 billion^3^ and predicted to exceed $15 billion by 2030 due to the increasing prevalence of risk factors including diabetes, obesity and metabolic syndrome.^4^

Metabolic testing is frequently utilized in the evaluation of recurrent urinary stone formers. The 24‐hour urine collection is recommended by the American Urological Association (AUA) and the European Association of Urology (EAU) to guide intervention strategies and prevent recurrence in high‐risk patients.^5^, ^6^ In this way, providers can use the test as part of long‐term follow‐up to monitor urinary constituents, such as calcium, oxalate, citrate and uric acid to assess recurrence risk and tailor interventions to the patient's risk profile. The test can be useful in first‐time or sporadic stone formers, particularly if predisposed to chronic kidney disease and/or metabolic bone disease.^7^ It can also be utilized for diagnostic purposes to potentially uncover stone aetiology and screen for metabolic diseases such as cystinuria and hyperoxaluria.^8^

Despite the recommendation of 24‐hour urine collections for stone management, the prevalence of testing remains low. One study found that only 7% of privately insured patients in the U.S. with high‐risk for stone recurrence received a 24‐hour urinalysis.^9^ Although the explanation is unclear, contributing factors may include limited guidelines regarding test performance, interpretation and frequency. There is no clear consensus in the current literature on the number of 24‐hour urine collections.^10^, ^11^, ^12^, ^13^ The AUA guidelines recommend one or two, with two being preferred, while the EAU guidelines recommend two for high‐risk stone formers.^5^, ^6^ However, the timing of these one or two collections is not specified. Currently, data is sparse on the role of serial 24‐hour urine collection tests done in stone formers at different time points. The purpose of this study was to determine whether serial 24‐hour urine collections done at three different time points reveal changes in urinary risk parameters for stone formation.

## 2 PATIENTS AND METHODS

After IRB approval, a retrospective review of a prospectively maintained database was performed, including 1832 urine collections from 688 patients collected over a 10‐year period. Of these, 225 patients had a minimum of 2‐year follow‐up and at least three 24‐hour urine collections. Exclusion criteria were age <18 years and follow‐up <2 years. 24‐hour urine collection samples were analysed by Litholink Labs (Labcorp, Itasca, IL). Following the initial 24‐hour urine collection, targeted personalized interventions were prescribed based on the results such as increasing fluids in those with urine output <2.5 L per day; low oxalate diet, pyridoxine and avoiding vitamin C supplements for patients with oxalate >38 [units]; moderate calcium diet, avoiding calcium supplements and addition of chlorthalidone for those with calcium >250 in men and >200 in women [units]; low animal protein diet and potassium citrate for those with hyperuricosuria; low salt diet for those with hypernatriuria; magnesium supplements for those with low magnesium; potassium citrate for those with low citrate and those requiring urine alkalinization.

Patient characteristics collected included age, gender and BMI. The following urine parameters were extracted from the database and analysed (Table 1): supersaturation of calcium oxalate (SSCaOx), supersaturation of calcium phosphate (SSCaP), supersaturation of uric acid (SSUA), calcium to creatinine ratio (CaCr), calcium per kilogram (CaKg), creatinine per kilogram (CrKg), creatinine (Cr), pH, potassium (K), calcium (Ca), oxalate (Ox), citrate (Cit), uric acid (UA), magnesium (Mg), sodium (Na) and phosphate (P).

**TABLE 1:** Comparison of change in mean values of 24‐hour urine parameters between each of three serial 24‐hour urine collections.

Test Parameter	Test 1 Mean	Test 2 Mean	Test 3 Mean	ANOVA (p < 0.05)	Test 1 vs Test 2 (p < 0.05)	Test 2 vs Test 3 (p < 0.05)	% Patients with Improvement (Test 1 to Test 2)	% Patients with Improvement (Test 2 to Test 3)
Volume (L)	2.193	2.636	2.696	<0.001	<0.001	0.060	65%	51%
SSCaOx	6.310	5.098	4.591	<0.001	<0.001	0.052	60%	55%
SSCaP	1.073	0.892	0.752	<0.001	0.006	0.014	57%	58%
SSUA	0.732	0.570	0.597	0.002	0.004	1.000	63%	46%
K (mmol)	58.47	62.41	61.30	0.034	0.018	1.000	57%	48%
CaCr	142.7	133.7	129.1	0.028	0.113	0.932	57%	58%
CaKg	2.501	2.371	2.283	0.044	0.332	0.794		
pH	6.207	6.264	6.232	0.291			57%	46%
Ca (mg)	198.2	188.3	184.1	0.066			53%	56%
Ox (mg)	38.99	39.16	38.12	0.666			47%	53%
Cit (mg)	550.9	581.4	575.4	0.161			55%	48%
UA (g)	0.570	0.570	0.565	0.867			50%	49%
Mg (mg)	100.3	102.8	101.8	0.611			54%	47%
Na (mmol)	164.8	168.5	160.1	0.229			48%	53%
P (g)	0.833	0.849	0.837	0.693			46%	50%

Differences in stone parameters among the three collection tests were initially analysed for statistical significance using repeated measures ANOVA with a Greenhouse–Geisser correction and post‐hoc analysis with Bonferroni correction. Parameters found to be significantly different in the initial analysis were subsequently analysed using a paired t‐test to compare each 24‐hour urine collection test to the next in order to determine which tests were significantly different from each other. Significance level was set at p < 0.05.

## 3 RESULTS

**FIGURE 1:**
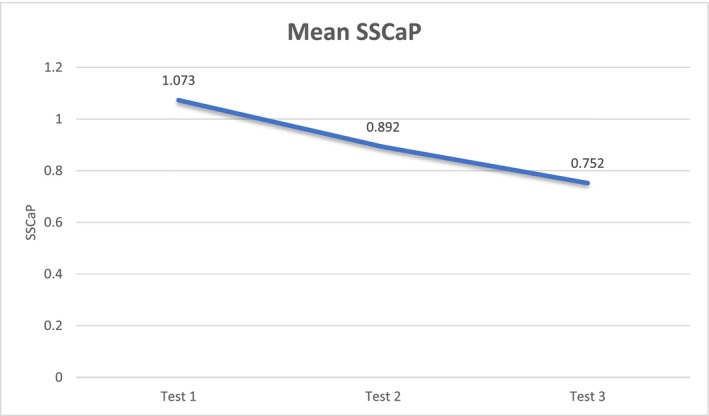
Change in mean supersaturation of CaP across three 24‐hour urine collections.

Among the 225 patients, 108 (48%) were female and 90 (40%) had a history of previous stone treatment. Mean age and BMI were 56.6 years and 28.5 kg/m^2^, respectively. Mean follow‐up time was 48.4 months. Based on stone analysis results from 420 stone specimens, the predominant stone type included calcium oxalate (62.2%), calcium phosphate (21.9%), uric acid (6.7%), struvite (1.9%), ammonium urate (1.0%), calcium carbonate (0.5%), cystine (0.5%) and 5.5% had two predominant stone types. A significant difference was observed among the three 24‐hour urine collection tests for urine volume, SSCaOx, SSCaP, SSUA, K, CaCr and CaKg (p < 0.05). Between the first and second 24‐hour urine collection tests, there was a significant improvement in urine volume, SSCaOx, SSCaP and SSUA (p < 0.05; Table 1). Although SSCaP was the only parameter that demonstrated significant improvement between the second and third 24‐hour urine collection test (Figure 1; p < 0.05), both urine volume (p = 0.06) and SSCaOx (p = 0.05) trended towards continued improvement. Cr did not vary >5% among the three urine collections, demonstrating high quality of urine collections.

When comparing the first and second 24‐hour urine collection tests, more than 50% of patients demonstrated improvements in urine volume, SSCaOx, SSCaP, SSUA and CaCr (Table 1). Between the second and third tests, improvements were observed in more than 50% of patients for urine volume, SSCaOx, CaP and CaCr. Although not statistically significant, at least 50% of patients had improvements in pH, Ca, Cit, UA and Mg when comparing the first and second tests and improvements in Ca, Ox, Na and P when comparing the second and third tests. Ca was the only parameter that consistently improved with each subsequent test.

## 4 DISCUSSION

Our results support the clinical utility of performing serial 24‐hour urine collection tests to continue to improve urine parameters in stone formers. Between the first and second 24‐hour urine collection tests, urine volume, SSCaOx, SSCaP, SSUA and CaCr significantly improved. Between the second and third 24‐hour urine collection tests, the SSCaP continued to significantly improve. Additionally, urine volume and SSCaOx continued to improve in 50% of patients between the second and third urine collections, nearly achieving statistical significance, which suggests that a larger sample size may have demonstrated significance. These data combined provide the first evidence in a relatively large cohort of patients that improvement in 24‐hour urinary stone parameters can be observed after obtaining three 24‐hour urine tests with at least 6 month intervals in between.

Our findings further support current AUA guidelines, which recommend that two 24‐hour urine collections are preferred over one.^5^ Other studies have supported the recommendation that two samples should be obtained. Nayan and associates analysed 24‐hour urine samples from 188 patients and concluded that two collections should be performed.^12^ Since the samples were collected on consecutive days, this allowed for assessment of day‐to‐day variability, but not variability over longer periods of time. In another study, Healy and colleagues retrospectively analysed 813 patients with two 24‐hour urine samples that were at least 10 days apart and concluded that two collections should be obtained to improve diagnostic yield and guide stone prevention strategies.^13^ Lastly, up to 50% variability in at least one major lithogenic factor between two consecutive 24‐hour urine collections was demonstrated in a study by Ennis and Asplin analysing 70 192 paired urine samples.^14^

While the benefit of three 24‐hour urine samples in the management of urolithiasis has not been fully elucidated, there is evidence that suggests potential for clinical utility. In a group of 75 idiopathic recurrent calcium stone formers, Hess et al. found that a greater number of abnormalities in major lithogenic risk factors such as volume, calcium and oxalate were detected after three 24‐hour urine collections compared to one or two.^15^ Similarly, Sun et al. found that three 24‐hour urine collections strongly correlated (r ≥ 0.8) with true long‐term urinary excretion of various minerals and electrolytes in a study of 3168 participants.^16^ Other studies that have analysed the utility of obtaining three 24‐hour urine collections have mainly been for nutritional assessment. McLean et al. performed a systematic review on 18 studies assessing the correlation between dietary sodium estimation by food frequency questionnaires and 24‐hour urinary excretion. They found that at least two or three 24‐hour urine collections, preferably obtained several months apart, are needed to accurately estimate usual sodium intake.^17^ Considering these data, there is potential clinical utility for obtaining three 24‐hour urine collections in the management of urolithiasis in selected high‐risk patients.

While a stone analysis is recommended by the AUA for patients newly diagnosed with a urinary stone,^5^ a 24‐hour urine collection may benefit some patient populations more than others. In particular, a first‐time or sporadic stone patient at risk for chronic kidney disease and/or metabolic bone disease should receive a 24‐hour urine test.^7^ Other special indications may include a history of multiple stones, family history, young age of onset, renal transplant, ureteral reimplantation or solitary kidney, chronic diarrhoea due to an underlying gastrointestinal condition and metabolic stones.^18^ These patients may benefit from receiving three or more 24‐hour urine collections. More studies, particularly prospective, are needed to determine the number of 24‐hour urine collections that should be obtained for optimal urinary stone management.

Besides metabolic evaluation, another utility of a 24‐hour urine collection is to guide treatment strategies and assess patient compliance to interventions. In a prospective multicentre study, Kocvara et al. followed 242 patients and found that stone recurrence and/or growth after three years occurred in only 7% of patients who received a personalized dietary regimen based on a 24‐hour urine collection compared to 23% of patients who had a general diet.^19^ Patients with a personalized diet were regularly followed with repeat collections and dietary changes. Following patients with serial 24‐hour urine collections may be clinically useful for determining treatment effectiveness, guiding treatment modifications and evaluating treatment compliance by monitoring for expected changes in urine composition following an intervention. Similarly, regular follow‐up visits have been shown to improve outcomes in various chronic diseases including chronic heart failure, weight loss after bariatric surgery and blood glucose control in type 2 diabetics.^20^, ^21^, ^22^ The benefit of regular follow‐ups may be linked to extrinsic motivation among patients to achieve desirable test results. The improvement in urinary parameters associated with 24‐hour urine tests could be mediated by the “stone clinic effect” which describes a phenomenon where fluid and dietary recommendations provided through long‐term follow‐up result in improved stone risk.^23^ Obtaining serial 24‐hour urine collections can help providers keep their patients accountable and potentially improve treatment compliance and effectiveness.

While current guidelines support the utilization of 24‐hour urine collection tests, the prevalence of testing is only 7.4%, and only 16.8% of patients who obtained the initial collection completed a follow‐up test.^9^ It is unclear exactly why the prevalence is low. One reason may be that clear guidelines are lacking on the long‐term management and follow‐up of stone patients.^24^ Other reasons include cost and burden to patients, errors in obtaining urine collections and uncertainty regarding interpretation of data and number of tests to perform.^25^ Out‐of‐pocket costs for Litholink, a common lab used for urine chemistries, can be as much as $700.^8^ Moreover, patients may view these tests as tedious and time‐consuming, resulting in a lack of compliance. Individuals who are most disciplined and strongly motivated to be on a long‐term preventive treatment regimen receive the most benefit from 24‐hour urine testing. For clinicians, identification of important information can be difficult due to the length and wording of the results.^18^ Interpretation is challenging as it involves connecting many different factors to correctly diagnose the patient and initiate proper treatment. As such, providers who are specifically trained in metabolic evaluation of urinary stones are most likely to utilize 24‐hour urine collections. Referring stone patients to a urologist or nephrologist may increase utilization of 24‐hour urine testing, as one study found that patients were three times more likely to complete a 24‐hour urine test if treated by one of these specialists.^9^

This study was limited by its retrospective and single‐institutional nature. Another limitation is missing data including dietary information and medical therapy. However, dietary information is subject to recall bias, and poor compliance to medical therapy for stones is common.^3^ Stone formation rates were not collected in this study, as the primary goal of this study was to evaluate changes in urinary parameters. Despite these limitations, this study utilized a prospectively maintained database to analyse data from a 10‐year period from a cohort of patients managed by a single provider consistently treated with the same management strategies. This is the first study to provide evidence that improvements in 24‐hour urinary stone parameters can be observed after obtaining three 24‐hour urine collections over an extended period of time.

## 5 CONCLUSIONS

Parameters important to stone formation such as urine volume, SSCaOx, SSCaP and SSUA were shown to significantly improve between the first and second 24‐hour urine tests, while SSCaP showed significant improvement between the second and third tests. There was continued improvement in many other urinary parameters between the second and third urine collections for half of the patients. Our data demonstrates that improvements in urinary stone parameters were significantly associated with obtaining at least three serial 24‐hour urine collections with at least 6‐month intervals in between. Long‐term follow‐up with 24‐hour urine collections can guide personalized treatment regimens, hold patients accountable and potentially improve treatment compliance and effectiveness.

## AUTHOR CONTRIBUTIONS

*Study conception*: D. Duane Baldwin, Akin S. Amasyali and Zhamshid Okhunov. *Data collection*: Daniel Jhang, Jason Groegler, Hyukje Sung, Matthew Buell, Jersey Castillo and Elizabeth A. Baldwin. *Statistical analysis*: Elizabeth A. Baldwin, Akin S. Amasyali and Mohamed Keheila. *Manuscript writing*: Daniel Jhang, Akin S. Amasyali, Zhamshid Okhunov and D. Duane Baldwin. *Manuscript review*: All authors. *Clinical supervision*: Akin S. Amasyali, Zhamshid Okhunov and D. Duane Baldwin.

## CONFLICT OF INTEREST STATEMENT

The authors have no conflicts of interest to disclose related to this manuscript.
